# Symptomatic intracerebral hemorrhage after non-emergency percutaneous coronary intervention: Incidence, risk factors, and association with cardiovascular outcomes

**DOI:** 10.3389/fcvm.2022.936498

**Published:** 2022-09-16

**Authors:** Mervyn Jun Rui Lim, Yilong Zheng, Rodney Yu-Hang Soh, Qi Xuan Joel Foo, Andie Hartanto Djohan, Vincent Nga Diong Weng, Jamie Sin-Ying Ho, Tseng Tsai Yeo, Hui-Wen Sim, Tiong-Cheng Yeo, Huay-Cheem Tan, Mark Yan-Yee Chan, Joshua Ping-Yun Loh, Ching-Hui Sia

**Affiliations:** ^1^Division of Neurosurgery, National University Health System, Singapore, Singapore; ^2^Yong Loo Lin School of Medicine, National University of Singapore, Singapore, Singapore; ^3^Department of Cardiology, National University Heart Centre, Singapore, Singapore; ^4^Academic Foundation Programme, North Middlesex University Hospital Trust, London, United Kingdom; ^5^Department of Medicine, Yong Loo Lin School of Medicine, National University of Singapore, Singapore, Singapore

**Keywords:** cerebrovascular disease, hemorrhagic stroke, epidemiology, cardiac catheterization, myocardial infarction

## Abstract

**Objective:**

To investigate the incidence, risk factors, and association with cardiovascular outcomes of patients who developed symptomatic intracerebral hemorrhage (ICH) after non-emergency percutaneous coronary intervention (PCI).

**Methods:**

We conducted a single-institution retrospective study of patients who developed symptomatic ICH after non-emergency PCI. To identify associations between clinical variables and outcomes, Cox-proportional hazards regression models were constructed. Outcomes analyzed include (1) all-cause mortality, (2) acute ischemic stroke (AIS) or transient ischemic attack (TIA), and (3) major adverse cardiovascular events (MACE).

**Results:**

A total of 1,732 patients were included in the analysis. The mean (±SD) age was 61.1 (±11.3) years, and 1,396 patients (80.6%) were male. The cumulative incidence of symptomatic ICH after non-emergency PCI was 1.3% (22 patients). Age, chronic kidney disease, and prior coronary artery bypass graft surgery were independently associated with a higher risk of ICH after PCI, while hyperlipidemia was independently associated with a lower risk of ICH after PCI. ICH after PCI was independently associated with a higher risk of all-cause mortality and AIS or TIA after PCI.

**Conclusion:**

Patients who are older, who have chronic kidney disease, and who have had prior coronary artery bypass graft surgery should be monitored for symptomatic ICH after non-emergency PCI.

## Introduction

Intracerebral hemorrhage (ICH) is an important complication after percutaneous coronary intervention (PCI) (1). The incidence of GUSTO (global utilization of streptokinase and tissue plasminogen activator for occluded coronary arteries) severe bleeding after PCI, as defined by ICH or any bleeding resulting in substantial hemodynamic compromise requiring treatment (2), was reported to be 0.2–2.5% (3, 4). ICH was associated with significant morbidity and mortality internationally (5, 6). ICH after PCI is particularly important as these patients were typically treated with dual antiplatelet therapy (DAPT) after PCI (7, 8), and prior antiplatelet therapy was associated with higher mortality rates among patients with ICH (9).

Despite the severity of ICH after PCI, to our knowledge, only one study investigated the incidence and outcomes of patients with *ICH only* after PCI (9). The study was limited by a relatively small sample size, did not assess independent risk factors for ICH, did not report long-term outcomes of ICH after PCI, and did not analyze the association between ICH after PCI and other cardiovascular outcomes of PCI (9). Thus, we aimed to investigate the incidence, risk factors, and association with cardiovascular outcomes of patients who developed symptomatic ICH after non-emergency PCI.

## Materials and methods

### Study population

This was a retrospective cross-sectional study of 1,732 consecutive patients who underwent semi-urgent or elective PCI between January 2014 to December 2015 at the National University Hospital, Singapore, a tertiary academic hospital. Semi-urgent PCI was defined as PCI done for non-ST segment elevation-acute coronary syndrome (NSTE-ACS), while elective PCI was defined as PCI done for a non-acute coronary syndrome (non-ACS) indication, such as an abnormal stress test. Institutional ethics approval was obtained from the local institutional review board before the commencement of the study, and a waiver of informed consent was granted since this study posed no more than minimal risks to participants.

### Data collection

Data was collected using a standardized data collection template from the electronic medical records of all patients. Data collected included demographics, cardiovascular risk factors, treatment variables, the occurrence of symptomatic ICH, death due to any reason, acute ischemic stroke (AIS) or transient ischemic attack (TIA), and major adverse cardiovascular events (MACE) after PCI. Demographics collected were age, sex, and ethnicity. Cardiovascular risk factors collected were smoking history (defined as never smoker, former smoker, and current smoker), history of acute myocardial infarction, hyperlipidemia, hypertension, diabetes mellitus, chronic kidney disease, AIS or TIA, atrial fibrillation, prior coronary artery bypass graft (CABG) surgery, and prior PCI.

Treatment variables related to the current PCI were indications for PCI (including type 1 non-ST segment elevation myocardial infarction (NSTEMI), positive exercise stress test, unstable angina, pre-operation assessment, previous positive computed tomography coronary angiography for ischemic heart disease, low ejection fraction, pre-transplant, type 2 myocardial infarction, arrhythmia, syncope, apical thrombus, and stabbing incident), whether DAPT (defined as any two out of Clopidogrel, Ticagrelor, Prasugrel, and Aspirin) was administered after PCI, and whether anticoagulation [including Warfarin and direct oral anticoagulants (including Rivaroxaban, Apixaban, or Dabigatran)] was administered after PCI.

ICH was diagnosed based on computed tomography (CT) scans of the brain for indications of (a) a decrease in the Glasgow Coma Scale score, (b) altered mental state, and (c) focal neurological deficits. All cases of ICH diagnosed in this study were symptomatic, and ICH was defined as spontaneous ICH, i.e., intracerebral hemorrhage that was not due to trauma or structural causes such as aneurysm, arteriovenous malformation, cavernous malformation, or tumor. Routine screening CT scans of the brain after PCI were not done. All-cause mortality was defined as mortality due to any reason. AIS or TIA after PCI was adjudicated by a stroke neurologist. MACE was defined as the occurrence of death due to any reason, myocardial infarction, chronic heart failure, or repeat revascularization of the target lesion. Clinical outcomes were obtained from the electronic medical records of the patients and were adjudicated by the treating physician.

### Statistical analysis

Baseline characteristics of patients with and without ICH were reported using mean and standard deviation for continuous variables, and count numbers and percentages for categorical variables. Hypothesis testing for continuous variables was conducted using the student's *t*-test. Hypothesis testing for categorical variables was conducted using the Pearson's *X*^2^ test, and the Fisher's exact test was used for variables that have less than five patients under any category (including ethnicity, smoking history, previous AIS or TIA, atrial fibrillation, indications of the current PCI, and the administration of anticoagulation after the current PCI). A *p*-value of <0.05 was taken to be statistically significant.

To investigate the independent risk factors for ICH after PCI, multiple Cox regression analysis was conducted. Variables that were statistically significant on univariate analysis and *a priori* risk factors of ICH [including sex, ethnicity, history of smoking, hypertension, diabetes mellitus, AIS or TIA, atrial fibrillation, and administration of anticoagulation therapy after PCI (10–12)] were specified as exposures in the model. ICH after PCI was specified as the dependent variable. A *p*-value of lesser than 0.05 was taken to be statistically significant, and the odds ratio and 95% confidence intervals of independent risk factors of ICH after PCI were reported. A test for the assumption of proportionality was performed, and the *p*-value for the global test for Schoenfeld residuals was 0.266, suggesting that the assumption of proportional hazards was fulfilled.

Time-to-event analysis was conducted to investigate the association between ICH after PCI and clinical outcomes. Time to the clinical outcome (i.e., all-cause mortality, AIS or TIA after PCI, or MACE after PCI) was defined as the duration in days between the date of the PCI and the date of the respective clinical outcomes. For patients who did not have the clinical outcome of interest, the follow-up time (defined as the duration in days between the date of the PCI and the date of the most recent clinic visit) was recorded instead. Kaplan-Meier curves for ICH after PCI against all-cause mortality, AIS or TIA after PCI, and MACE after PCI were plotted. Hypothesis testing using the log-rank test was conducted. One patient who had missing data on the indication of the current PCI was excluded from all the analyses. Seven patients who had missing data on all-cause mortality were excluded from the analysis for all-cause mortality. Nine patients who had missing data on AIS or TIA after PCI were excluded from the analysis for AIS or TIA after PCI.

Cox-proportional hazards models were built to investigate the association between ICH after PCI and all-cause mortality, AIS or TIA after PCI, and MACE after PCI. For each of the outcomes, we adjusted for known confounders (13–21) in three separate models. In the first model, we adjusted for demographics, including age, sex, and ethnicity. In the second model, we adjusted for the variables in the first model as well as cardiovascular risk factors, including smoking history, history of acute myocardial infarction, hyperlipidemia, hypertension, diabetes mellitus, chronic kidney disease, previous AIS or TIA, atrial fibrillation, prior CABG surgery, and prior PCI. In the third model, we adjusted for the variables in the second model as well as treatment variables related to the current PCI, including the indications of the current PCI, administration of DAPT after the current PCI, and administration of anticoagulation therapy after the current PCI. A *p*-value of lesser than 0.05 was taken to be statistically significant, and the hazards ratio and 95% confidence intervals were reported. On testing for the assumption of proportionality, the *p*-value for the global test for Schoenfeld residuals was above 0.050 for all the models, suggesting that the assumption of proportional hazards was fulfilled for all models. All data analyses were conducted using R Studio Version 1.2.5042.

## Results

### Incidence of ICH after PCI

A total of 1,732 patients were included in the analysis. Twenty-two patients (1.3%) had ICH after PCI, of which 9 patients had symptomatic ICH within 12 months after PCI, while 13 patients had symptomatic ICH more than 12 months after PCI. Symptomatic ICH occurred at a median (IQR) of 1.31 (0.13–3.00) years after PCI. The mean (±SD) duration of follow-up was 3.71 (±0.97) years. The mean (±SD) age of the study population was 61.1 (±11.3) years, and 1,396 patients (80.6%) were male. On univariate analysis, age (*p* = 0.002), history of acute myocardial infarction (*p* = 0.013), hyperlipidemia (*p* = 0.020), chronic kidney disease (*p* = 0.006), prior CABG surgery (*p* = 0.010), administration of DAPT after PCI (*p* = 0.011), all-cause mortality (*p* = 0.002), AIS or TIA after PCI (*p* < 0.001), and MACE after PCI (*p* < 0.001) were associated with ICH after PCI. Baseline characteristics, clinical variables, and clinical outcomes of patients with and without ICH after PCI were presented in Table 1.

**Table 1:** Baseline characteristics of patients with and without intracerebral hemorrhage after non-emergency percutaneous coronary intervention.

**Variable**	**Intracerebral hemorrhage (*n* = 22, 1.3%)**	**No intracerebral hemorrhage^1^ (*n* = 1,710, 98.7%)**	**Total (*n* = 1,732)**	***P*-value**
Age; mean (SD)	68.5 (9.9)	61.0 (11.3)	61.1 (11.3)	**0.002**
Sex, Male; *n* (%)	18 (81.8)	1378 (80.6)	1396 (80.6)	1.000
**Ethnicity;** ***n*** **(%)**				0.736
Chinese	15 (68.2)	1,018 (59.5)	1,033 (59.6)	
Malay	3 (13.6)	283 (16.5)	286 (16.5)	
Indian	3 (13.6)	217 (12.7)	220 (12.7)	
Others	1 (4.5)	192 (11.2)	193 (11.1)	
**Smoking history;** ***n*** **(%)**				0.846
Current smoker	7 (31.8)	501 (29.4)	508 (29.3)	
Former smoker	4 (18.2)	274 (16.0)	278 (16.1)	
Never smoker	11 (50.0)	935 (54.7)	946 (54.6)	
Acute myocardial infarction; *n* (%)	20 (90.9)	1,073 (62.7)	1,093 (63.1)	**0.013**
Hyperlipidemia; *n* (%)	12 (54.5)	1,328 (77.7)	1,340 (77.4)	**0.020**
Hypertension; *n* (%)	18 (81.8)	1,210 (70.8)	1,228 (70.9)	0.369
Diabetes mellitus; *n* (%)	7 (31.8)	723 (42.3)	730 (42.1)	0.441
Chronic kidney disease; *n* (%)	8 (36.4)	232 (13.6)	240 (13.9)	**0.006**
Previous acute ischemic stroke or transient ischemic attack; *n* (%)	2 (9.1)	142 (8.3)	144 (8.3)	0.704
Atrial fibrillation; *n* (%)	4 (18.2)	116 (6.8)	120 (6.9)	0.061
Prior coronary artery bypass graft surgery; *n* (%)	5 (22.7)	112 (6.5)	117 (6.8)	**0.010**
Prior PCI; *n* (%)	5 (22.7)	525 (30.7)	530 (30.6)	0.558
**Indications of the current PCI;** ***n*** **(%)**				0.434
Type 1 non-ST segment elevation myocardial infarction	18 (81.8)	937 (54.8)	955 (55.2)	
Positive exercise stress test	0 (0.0)	246 (14.4)	246 (14.2)	
Unstable angina	1 (4.5)	205 (12.0)	206 (11.9)	
Pre-operation assessment	1 (4.5)	152 (8.9)	152 (8.8)	
Previous positive computed tomography coronary angiography for ischemic heart disease	1 (4.5)	70 (4.1)	71 (4.1)	
Low ejection fraction	1 (4.5)	57 (3.3)	58 (3.4)	
Pre-transplant	0 (0.0)	28 (1.6)	28 (1.6)	
Type 2 myocardial infarction	0 (0.0)	10 (0.6)	10 (0.6)	
Others^2^	0 (0.0)	4 (0.3)	5 (0.3)	
Administration of dual antiplatelet therapy after PCI; *n* (%)	16 (72.7)	1,555 (91.4)	1,571 (91.1)	**0.011**
Administration of anticoagulation therapy (Warfarin and DOACs) after PCI; *n* (%)	3 (13.6)	83 (4.9)	86 (5.0)	0.092
All-cause mortality; *n* (%)	8 (36.4)	209 (12.3)	217 (12.6)	**0.002**
Acute ischemic stroke or transient ischemic attack after PCI; *n* (%)	10 (45.5)	60 (3.5)	70 (4.1)	**<0.001**
Major adverse cardiac events after PCI; *n* (%)	11 (50.0)	267 (15.7)	278 (16.1)	**<0.001**

1 Among the patients with no intracerebral hemorrhage after PCI, there was 1 patient who had missing data on the indication of the current PCI, 7 patients who had missing data on all-cause mortality, and 9 patients who had missing data on acute ischemic stroke or transient ischemic attack after PCI.

2 These include arrhythmia, syncope, apical thrombus, and stabbing incident.

SD, standard deviation; PCI, percutaneous coronary intervention; DOACs, direct oral anticoagulants.

### Independent risk and protective factors associated with ICH

On Cox regression analysis (Table 2), age (HR = 1.06; 95% CI = 1.01–1.11; *p* = 0.009), chronic kidney disease (HR = 3.86; 95% CI = 1.40–10.7; *p* = 0.009), and prior CABG surgery (HR = 3.33; 95% CI = 1.11–11.2; *p* = 0.032) were associated with a higher risk of ICH after PCI, while hyperlipidemia was associated with a lower risk of ICH after PCI (HR = 0.17; 95% CI = 0.06–0.47; *p* < 0.001).

**Table 2:** Multiple Cox regression model showing the independent risk factors for intracerebral hemorrhage after non-emergency percutaneous coronary intervention.

**Variable^1^**	**HR**	**95% CI**	***P*-value**
Age	1.06	1.01–1.11	0.009
Chronic kidney disease	3.86	1.40–10.7	0.009
Prior coronary artery bypass graft surgery	3.33	1.11–11.2	0.032
Hyperlipidemia	0.17	0.06–0.47	<0.001

1 Risk factors included in the model include demographics (including age, sex, and ethnicity), cardiovascular risk factors (including history of smoking, acute myocardial infarction, hyperlipidemia, hypertension, diabetes mellitus, chronic kidney disease, acute ischemic stroke or transient ischemic attack, atrial fibrillation, and coronary artery bypass graft surgery), administration of dual antiplatelet therapy after the current PCI, and administration of anticoagulation therapy after the current PCI.

HR, hazard ratio; CI, confidence interval; PCI, percutaneous coronary intervention.

### Association between ICH and clinical outcomes after PCI

**Figure 1:**
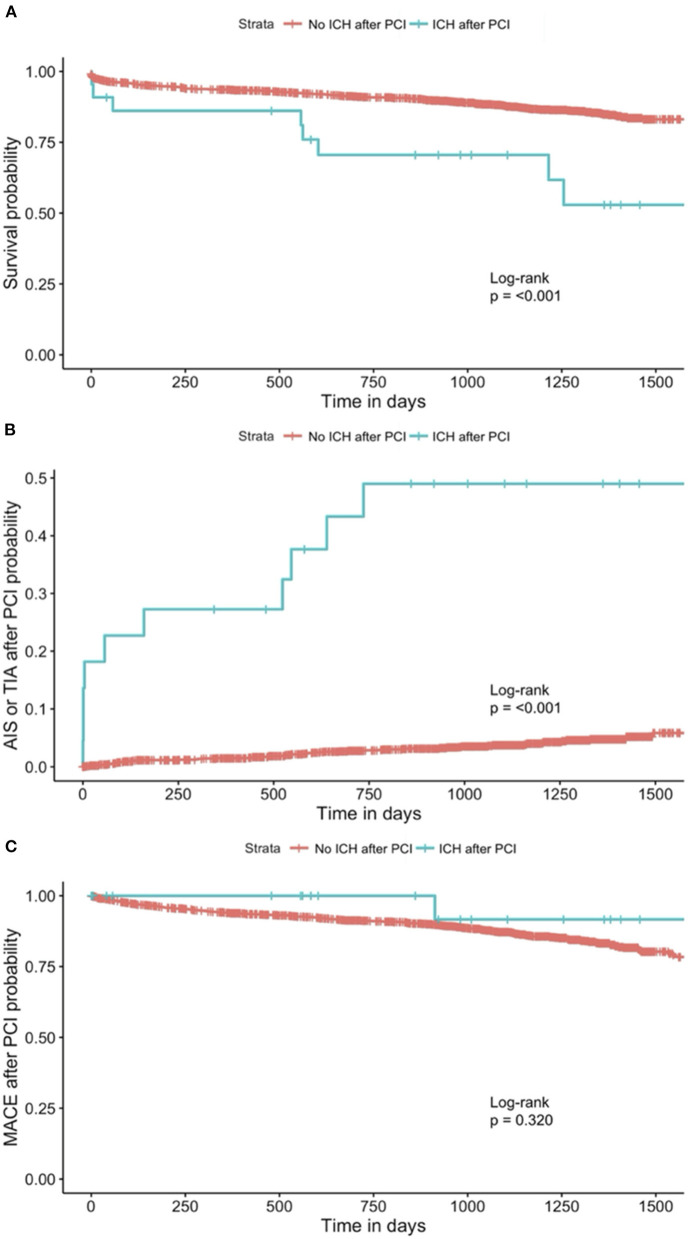
Kaplan-Meier curve for **(A)** all-cause mortality^1, 2^, **(B)** AIS or TIA after PCI^1, 3^, and **(C)** MACE after PCI^1^, stratified by patients with and without ICH after PCI. ^1^The one patient who had missing data on the indication of the current PCI was excluded from the analysis. ^2^The seven patients who had missing data on all-cause mortality were excluded from the analysis. ^3^The nine patients who had missing data on AIS or TIA after PCI were excluded from the analysis. AIS, acute ischemic stroke; TIA, transient ischemic attack; MACE, major adverse cardiovascular events; PCI, percutaneous coronary intervention; ICH, intracerebral hemorrhage.

In our study population, 36.4% of patients who had ICH (eight patients) died, while only 12.3% of patients who did not have ICH (209 patients) died (Table 1). On Cox-proportional hazards regression analysis (Table 3), ICH after PCI was associated with a higher risk of all-cause mortality (HR = 2.46, 95% CI = 1.21–5.00; *p* = 0.013) after adjusting for demographics, but not after adjusting for cardiovascular risk factors and treatment variables related to the current PCI. ICH after PCI was also associated with AIS or TIA after PCI (HR = 11.4; 95% CI = 5.39–24.3; *p* < 0.001) after adjusting for demographics, cardiovascular risk factors, and treatment variables related to the current PCI. There was no significant association between ICH and MACE after PCI. The Kaplan-Meier curves for ICH after PCI against all-cause mortality, AIS or TIA after PCI, and MACE after PCI and results of the log-rank test were presented in Figure 1.

**Table 3:** Cox-proportional hazards models showing the association between intracerebral hemorrhage after non-emergency percutaneous coronary intervention and clinical outcomes.

**Association between ICH and clinical Outcomes**	**Model I^a^**			**Model II^b^**			**Model III^c^**		
	**HR**	**95% CI**	***P*-value**	**HR**	**95% CI**	***P*-value**	**HR**	**95% CI**	***P*-value**
All-cause mortality^1^^,^ ^2^	2.46	1.21–5.00	**0.013**	1.48	0.72–3.05	0.286	1.40	0.67–2.92	0.365
AIS or TIA after PCI^1^^,^ ^3^	15.2	7.62–30.3	**<0.001**	12.3	5.97–25.6	**<0.001**	11.4	5.39–24.3	**<0.001**
MACE after PCI^1^	3.32	0.46–23.9	0.232	3.75	0.52–27.2	0.191	3.978	0.53–29.6	0.178

a Model I: Adjusted for demographics, including age, sex, and ethnicity.

b Model II: Adjusted for variables in Model I and cardiovascular risk factors, including smoking history, history of acute myocardial infarction, hyperlipidemia, hypertension, diabetes mellitus, chronic kidney disease, AIS or TIA, atrial fibrillation, prior coronary artery bypass graft surgery, and prior PCI.

c Model III: Adjusted for variables in Model II and treatment variables related to the current PCI, including the indications of the current PCI, administration of dual antiplatelet therapy after the current PCI, and administration of anticoagulation therapy after the current PCI.

1 The one patient who had missing data on the indication of the current PCI was excluded from the analysis.

2 The seven patients who had missing data on all-cause mortality were excluded from the analysis.

3 The nine patients who had missing data on AIS or TIA after PCI were excluded from the analysis.

ICH, intracerebral hemorrhage; HR, hazards ratio; CI, confidence interval; AIS, acute ischemic stroke; TIA, transient ischemic attack; PCI, percutaneous coronary intervention; MACE, major adverse cardiovascular events.

The bold values indicate statistical significance.

## Discussion

In our study, the cumulative incidence of ICH after non-emergency PCI over the study period (January 2014 to December 2015) was 1.3%. This incidence is within the range of incidences of GUSTO severe bleeding after PCI, which was reported to be 0.2–2.5% (3, 4). Aside from ICH, GUSTO severe bleeding also includes any type of bleeding resulting in substantial hemodynamic compromise requiring treatment. Therefore, the rate of ICH is likely to be lower than 0.2–2.5%. The only study that reported the incidence of only ICH after PCI reported an incidence of 15.0% (9). However, that study included only patients who had an ischemic stroke before PCI. We hypothesize that the incidence of ICH in our cohort was lower as the patients in our cohort may have less severe cardiovascular risk factors than the cohort of patients included in the study by Schmidbauer et al. (9), who all had an acute ischemic stroke before the emergency coronary catheterization for acute myocardial infarction during the same hospital admission.

We identified age, chronic kidney disease, and prior CABG surgery as independent risk factors for ICH, and hyperlipidemia as an independent protective factor for ICH after non-emergency PCI. To our knowledge, there were no studies from the international literature that studied the risk factors for post-PCI ICH specifically. Age (22, 23) and chronic kidney disease (23) were reported as risk factors for intracranial hemorrhage after PCI in the existing literature. Reports of ICH after CABG surgery in the literature were limited to case reports (24). We hypothesize that patients with prior CABG were at higher risk of ICH because of cerebral microbleed formation post-CABG (25), which has been associated with a higher risk of ICH (26).

In addition, our findings support data from the international literature that hyperlipidemia was associated with a lower risk of ICH after PCI. In a meta-analysis of 23 prospective studies consisting of 1,430,141 participants, Wang et al. found that hyperlipidemia, specifically hypercholesterolemia, was inversely associated with the risk of hemorrhagic stroke (27). Hypercholesterolemia may play a role in suppressing necrosis of the tunica media (28), thereby lowering the risk of microaneurysm formation and the risk of ICH (29). However, hyperlipidemia is a known risk factor for ischemic stroke and other cardiovascular diseases such as ischemic heart disease. Recent meta-analyses have also shown no association between statin therapy and ICH (30–32). A meta-analysis of 61 prospective studies showed that statin therapy significantly reduced not only the rate of coronary events but also total stroke rates (33). Thus, existing international guidelines recommend statin therapy for patients with coronary artery disease (34).

Our study also found a significant association between ICH after PCI and all-cause mortality and AIS or TIA after PCI. Major bleeding events after PCI were associated with a higher risk of mortality. In a meta-analysis of 42 studies consisting of 533 333 patients, Kwok et al. (1) showed that major bleeding post-PCI was independently associated with a higher risk of all-cause mortality (OR = 3.31; 95% CI = 2.86–3.82) and MACE (OR = 3.90; 95% CI = 3.26–4.64). However, ICH was not analyzed independently but was instead grouped under major bleeding, a composite variable. The association between ICH alone after PCI and all-cause mortality was not studied, and therefore it is not clear whether an association between ICH after PCI and all-cause mortality exists. To our knowledge, there was only one study that examined the outcomes of ICH alone after PCI. Schmidbauer et al. (9) reported no intrahospital mortalities from ICH in their retrospective cohort of 20 patients who underwent cardiac catheterization for acute myocardial infarction after ischemic stroke. While this study examined the outcomes of patients with ICH alone after PCI, only short-term outcomes of the patients were reported. The small sample size of the cohort also limited the robustness of the analysis.

The strengths of our study were that it included a large cohort of patients who underwent non-emergency PCI, had a clear definition of symptomatic ICH, and there was a long duration of follow-up. Our study had several limitations. First, our study was limited by the small numbers of patients with ICH which may have limited the power of the study to detect other significant risk factors and the possibility of unmeasured confounders in the analysis. Second, our study included only patients who underwent non-emergency PCI. We hypothesize that the incidence of symptomatic ICH may be higher if patients with emergency PCI were included in the analysis (9). Third, our study included only patients from a single institution, and therefore the results may not be generalizable to other populations. Fourth, most patients included in our study were Asians. As the rates of intracerebral hemorrhage were reported to be higher among Asians than patients of other ethnicities (5), the findings from our study may not be extrapolated to populations consisting of predominantly non-Asian patients. Fifth, specific data about the ICH such as the volume of the ICH and the treatment for the ICH were not collected and analyzed. Lastly, the retrospective nature of this study allowed us to only study association and not causation between the clinical variables and outcomes.

## Conclusion

The cumulative incidence of symptomatic ICH after non-emergency PCI over the study period (January 2014 to December 2015) was 1.3%. Age, chronic kidney disease, and prior coronary artery bypass graft surgery were independently associated with a higher risk of ICH after PCI, while hyperlipidemia was independently associated with a lower risk of ICH after PCI. ICH after PCI was independently associated with a higher risk of all-cause mortality and AIS or TIA after PCI. Our study suggests that patients who are older, who have chronic kidney disease, and who have had prior coronary artery bypass graft surgery should be monitored closely for symptomatic ICH after PCI.

## Data availability statement

The raw data supporting the conclusions of this article will be made available by the authors, without undue reservation.

## Ethics statement

The studies involving human participants were reviewed and approved by Domain Specific Review Board, National Healthcare Group. Written informed consent for participation was not required for this study in accordance with the national legislation and the institutional requirements.

## Author contributions

ML and YZ contributed equally to the writing of the manuscript. All authors contributed significantly to the data collection and critical review of the manuscript.

## Conflict of interest

The authors declare that the research was conducted in the absence of any commercial or financial relationships that could be construed as a potential conflict of interest.

## Publisher's note

All claims expressed in this article are solely those of the authors and do not necessarily represent those of their affiliated organizations, or those of the publisher, the editors and the reviewers. Any product that may be evaluated in this article, or claim that may be made by its manufacturer, is not guaranteed or endorsed by the publisher.
